# Speciation Pattern and Process in the California Coastal Dune Endemic Trapdoor Spider *Aptostichus simus* (Mygalomorphae: Euctenizidae) and Description of a New Cryptic Species

**DOI:** 10.1002/ece3.72346

**Published:** 2025-10-22

**Authors:** Emma E. Jochim, James Starrett, Hanna R. Briggs, Jason E. Bond

**Affiliations:** ^1^ University of California, Davis Davis California USA

**Keywords:** Araneae, continuous character traits, integrative taxonomy, phylogenomics, species delimitation, ultraconserved elements

## Abstract

The application of genomic and sub‐genomic data in species delimitation has facilitated the discovery of cryptic species. As the name implies, cryptic species are difficult, if not impossible, to distinguish based on morphology alone. The integrative species delimitation process employed herein comprises three steps: species discovery, species validation, and species description. Phylogenetic analysis of sub‐genomic data revealed three major lineages within the trapdoor spider *Aptostichus simus.* These lineages identified candidate species that were then tested using further genetic and morphological analyses. The species validation step supported the discovery of a novel cryptic species, *A. ramirezae* sp. nov., and potential incipient species. *Aptostichus simus* and *A. ramirezae* sp. nov., are endemic to coastal dune habitats in California and Baja California, which face many threats such as erosion, human development, habitat fragmentation, coastal squeeze, and sea level rise. Understanding the patterns of genetic diversity in these species is crucial for informing conservation efforts of both the animals and habitat in which they live.

## Graphical abstract

The integrative species delimitation process employed herein comprises three steps: species discovery, species validation, and species description. Phylogenetic analysis of sub‐genomic data revealed three major lineages within the trapdoor spider *Aptostichus simus* . The species validation step supported the discovery of a novel cryptic species, *A. ramirezae* sp. nov., and potential incipient species.

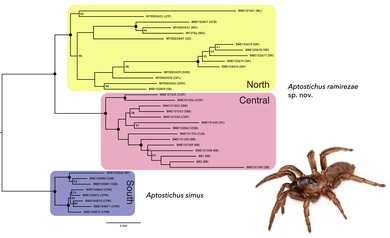

## 1 Introduction

Species can be conceptualized as separately evolving metapopulation lineages (De Queiroz 2005, 2007). Although this generalized lineage species concept has been widely referenced, its application as an aid to formal delimitation is limited owing to disagreement as to which secondary defining properties are required to delimit species. Because species arise as the result of the speciation process, the act of delimiting newly recognized species should not necessarily be decoupled from deciphering those processes. However, because speciation is continuous, it is often unclear at what point lineages should be considered distinct species (De Queiroz 2007; Stankowski and Ravinet 2021). Taxonomic decisions can be further complicated if species boundaries are evaluated as testable hypotheses while also recognizing the need for accurate description and character diagnosis (Dayrat 2005). An integrative approach incorporates evidence from multiple sources to obtain a holistic view of what constitutes a species (Dayrat 2005). It considers the processes that generate molecular divergence uncovered at the early stages of species discovery and evaluates how these have affected other characters (e.g., morphology and behavior) used to test species hypotheses (Bond and Stockman 2008; Opatova et al. 2023; Padial et al. 2010).

Species delimitation has three steps: species discovery, species validation, and species description (Carstens et al. 2013; Opatova et al. 2023; Wüster et al. 2024). The species discovery stage typically employs molecular data to uncover divergence that reflects separately evolving metapopulation lineages. These lineages are considered “candidate species” as they potentially represent distinct species not formally described. During the validation step, other evidence is used to attempt to falsify these hypotheses generated during discovery. This additional evidence can be morphological, behavioral, ecological, and/or other molecular (e.g., genomic vs. mitochondrial). If a candidate species hypothesis is supported by data analyzed during validation, then it can, and should, be formally described (Jörger and Schrödl 2013). The final step of nominal species description should not be overlooked.

Because nominal species require secondary defining properties that are diagnostic, species boundaries can be particularly difficult to identify and formally describe in groups with morphological homogeneity (Jörger and Schrödl 2013). Cryptic species, taxa that are morphologically indistinguishable but described as a single species (Mayr 1963; Bickford et al. 2007), lead to diversity underestimation (Funk et al. 2012; Hebert et al. 2004; Jörger and Schrödl 2013; Nadler and De León 2011; Nygren 2014) and downstream risks of crypto‐extinction, where a species goes extinct before discovery and description (Giam et al. 2012). Conversely, in groups with low dispersal ability and concomitant microallopatric population structuring, coalescent based delineation approaches tend to overestimate diversity (see Supplemental Methods; Bond and Stockman 2008; Sukumaran and Knowles 2017). Using genomic or sub‐genomic data rather than only one or a few loci, scientists are discovering cryptic species at a rapid rate (Bickford et al. 2007), as observed for some key spider groups (Bond and Stockman 2008; Ciaccio et al. 2022; Ferretti et al. 2024; Hedin et al. 2015; Opatova et al. 2023; Rix et al. 2017; Satler et al. 2013; Starrett et al. 2024).

The spider infraorder Mygalomorphae (Araneae) includes fossorial, long‐lived taxa such as tarantulas and trapdoor spiders. These spiders often exhibit high levels of morphological conservation within and between taxa, and morphological homogeneity is believed to be the result of convergent evolution (Wilson et al. 2023). Specifically, in trapdoor spiders the construction of silk‐lined, subterranean burrows with a camouflaged flap at the entrance is thought to have led to extremely conserved somatic morphologies, e.g., spinneret shape, coloration, chaetotaxy, eye arrangement, even between distantly related groups (Wilson et al. 2023). Evaluating whether these morphologically indistinct groups are harboring cryptic species requires approaches integrating multiple data types (e.g., behavioral, ecological, genetic, etc.). Integrative methods are prominently employed to delimit mygalomorph species (Bond 2012; Bond and Stockman 2008; Ferretti et al. 2024; Hedin et al. 2019; Leavitt et al. 2015; Monjaraz‐Ruedas et al. 2023; De Montes Oca et al. 2016; Newton et al. 2020, 2023).

*Aptostichus simus* Chamberlin, 1917 is a North American trapdoor spider endemic to coastal dunes ranging from Baja California, Mexico, to Monterey Bay, California, with populations also known from two Northern California Channel Islands. It is one of 37 species of *Aptostichus* found largely in the California Floristic Province biodiversity hotspot (Bond 2012; Myers et al. 2000). Although *A. simus* is widespread latitudinally, it is longitudinally very restricted due to high habitat specificity. These spiders build deep silk‐lined burrows with a cryptic trapdoor covered with sand and are commonly found at the base of native dune vegetation (Bond 2012; Bond et al. 2001). Other *Aptostichus* species build burrows in more compact and rocky substrates. Consequently, *A. simus* populations are thought to be extremely sensitive to habitat degradation (Bond et al. 2001).

Two previous studies have explored *A. simus* populations. First, Ramirez and Froehlig (1997) used allozymes to examine gene flow and population subdivision across nine *A. simus* populations from Point Dume northward into Southern Ventura County. Thirteen loci were fixed and thus were considered genetically homogeneous and in Hardy–Weinberg equilibrium. Their study included a subset of populations, which did not include the type locality at Silver Strand State Beach in San Diego Co., and allozyme genetic markers that, although widely used at the time, can underestimate population level diversity (Hoy 2019). Second, Bond et al. (2001) reexamined molecular divergence in *A. simus* using mitochondrial 16S rRNA data and widely sampled populations from across the entirety of the *A. simus* range. This phylogeographic study uncovered deep and ancient molecular divergence not reflected in morphological differences. Bond et al. estimated that the earliest diverging mitochondrial haplotype, those in San Diego County, separated from the more northern populations 2–3 million years ago. Additionally, they estimated very high pairwise levels of haplotype sequence divergence relative to other published population studies (see Bond et al. 2001). Despite high genetic divergence and long temporal discontinuity, they conservatively did not further split *A. simus* into multiple species owing to the study's reliance on mitochondrial data and insufficient male sample sizes (Bond et al. 2001). The results of Bond et al. (2001) contrast with those of Ramirez and Froehlig (1997), likely a result of the data types, mitochondrial DNA and allozymes, respectively, and sample sizes used in each study. Although both works were indicative of interesting population dynamics with the latter work potentially indicating speciation, no further work on this group has since been undertaken.

The principal aim of this study is to reexamine species boundaries across *Aptostichus simus* . We employed an integrative framework to test the hypothesis that *A. simus* comprises multiple species. We generated genomic scale data to infer the phylogeny of *A. simus* and then investigated morphological and population‐level differences to determine if these factors are reflected in the genetic groups. Large genomic‐scale datasets are key to answering a variety of evolutionary questions and have been used in many mygalomorph phylogeographic (Hamilton et al. 2011; Newton et al. 2023), conservation (Ferretti et al. 2014; Marsh et al. 2023), and systematic (Hedin et al. 2019; Monjaraz‐Ruedas et al. 2023; Opatova et al. 2020; Starrett et al. 2024) studies. We apply Templeton's Cohesion Species Concept (CSC; Templeton 1989) by testing for genetic and phenotypic interchangeability and conclude that *A. simus* as currently described comprises a cryptic species. *Aptostichus ramirezae* sp. nov. from Moss Landing State Beach; it is formally described based on these results.

## 2 Materials and Methods

### 2.1 Sampling

Specimens were collected from Baja California Norte to Monterey County (Bond 2012), including two new localities (S1; see also Bond 2012). Two outgroup individuals were collected from Kern County and are a currently undescribed species but are the closest known relatives of *A. simus*.

### 2.2 Sequence Capture Ultraconserved Elements

Ultraconserved element (UCE) data was generated following Faircloth et al. (2012) with modifications from Hedin et al. (2019), Kulkarni et al. (2020), and Starrett et al. (2017). We extracted DNA from spider legs using the Blood and Tissue Qiagen DNeasy kit. DNA quantity and quality were checked using Qubit 3.0 Fluorometer (Life Technologies) and agarose gel. DNA concentrated at 250 ng was fragmented to 200–1000 bp using an ultrasonicator (Covaris) with the following settings: 105 watt Peak Index Power, 5% Duty Factor, 200 Cycles Per Burst, and ~120 s treatment time. UCE libraries were generated with the KAPA Hyperprep kit (Roche) with universal adapters and iTru5/7 barcodes (Glenn et al. 2019; BadDNA@UGA). Libraries were hybridized at 60°C for 24 h to the Spider probe set (Kulkarni et al. 2020) following the version 4 chemistry protocol (Arbor Biosciences). Hybridization enriched libraries were sequenced with 150 bp paired‐end reads on the HiSeq4K (Illumina) or AVITI (Element Biosciences) at the UC Davis DNA Technologies Core. Additional samples were processed by RAPID Genomics (Gainesville, FL).

Raw reads were filtered and trimmed using Illumiprocessor (B. C. Faircloth 2013) and Trimmomatic (Bolger et al. 2014) in PHYLUCE v1.7.1 (B. C. Faircloth 2016). Cleaned paired‐end and single‐end reads were assembled using SPAdes v3.14.1 (Prjibelski et al. 2020). Contigs were matched to the blended (Spider + Arachnid) UCE probe list (Maddison et al. 2020). MAFFT v7.475 (Katoh and Standley 2013) was used to align locus datasets with occupancy minimums of 75%, 85%, and 95%. After the major ingroup relationships were established, i.e., which ingroup clade is sister to the rest, outgroups and individuals with missing data were removed from the dataset. Reads for remaining individuals (13 juveniles, 23 females, and one male; *n* = 37) were internally trimmed with trimAL (Capella‐Gutierrez et al. 2009), mapped to assembled contigs, and low‐depth and low‐quality base calls were removed using mapping and correction workflows in PHYLUCE v1.7.3 (B. C. Faircloth 2016).

### 2.3 Phylogenetic Analysis

Phylogenies were inferred from concatenated alignments of three data sets (minimum of 75%, 85%, and 95% locus occupancy) using maximum likelihood inference in IQ‐TREE v2.1.2 (Minh et al. 2020). The best locus partition and substitution model were selected using ModelFinder (Kalyaanamoorthy et al. 2017) implemented in IQ‐TREE, and support values were inferred from 1000 pseudoreplicates using ultrafast bootstrapping (Hoang et al. 2018; Minh et al. 2013). Coalescence‐based species trees were generated with ASTRAL‐III (Zhang et al. 2018) using gene trees from the 75% locus occupancy datasets (IQ‐TREE analyses). Multispecies coalescent bootstrap resampling was run with 100 pseudoreplicates in ASTRAL v5.7.8 (Simmons et al. 2019). 50%, 70%, and 100% consensus species trees were generated using the consensus tree builder function in Geneious Prime (Geneious Prime 2024.0.7 [https://www.geneious.com]).

### 2.4 Population Analyses

Unlinked single nucleotide polymorphism (SNP) datasets were generated for 32 ingroup individuals (excluding individuals from Santa Rosa Island and Baja California due to high amounts of missing data). Reads were mapped against corresponding scaffolds with Burrows‐Wheeler Aligner (BWA) (Li and Durbin 2010) implemented in PHYLUCE and using the default parameters. Alignments were phased and filtered to contain sites with at least 75% of taxa represented. Alignments were screened for unlinked SNPs using snps_from_uce_alignments.py (Andermann et al. 2018) and three sets of randomly chosen unlinked SNPs (RANDSNP1–3) were generated. FASTA files containing phased SNP data were converted to “one‐hot encoding” and a genetic clustering analysis, Variational AutoEncoder (VAE), was performed to visualize population clustering (Derkarabetian et al. 2019). The RANDSNP1 dataset was used to estimate nucleotide diversity (π) for the three clades in the R package *pegas* (Paradis 2010).

To determine the level of genetic differentiation between clades, we calculated Hedrick's G_ST_ (G'_ST_) using the SNP datasets comprising 32 individuals. This metric was chosen because it is standardized and has a range from 0 to 1, regardless of effective population sizes and levels of genetic variation between loci (Hedrick 2005). Jost's D was also calculated to measure allelic differentiation between the three clades (Jost 2008). Files containing SNP data were converted to genind objects in R using the package *adegenet* (Jombart and Ahmed 2011), and G'_ST_ and Jost's D were calculated using the R package *mmod* v1.3.3 (Winter 2012).

Admixture coefficients were estimated using sparse Non‐Negative Matrix Factorization (sNMF) algorithms in the R package LEA (Frichot and François 2015) with α = 100 and *K* values ranging from 1 to 10 for all SNP datasets. Cross‐entropy validation was performed to select the optimal *K* value. sNMF Q‐matrices were visualized using the barchart function in LEA.

A variant call format (VCF) file for all ingroup and outgroup individuals was generated from cleaned UCE reads following the methods in Monjaraz‐Ruedas et al. (2024). Using VCFtools v0.1.14 (Danecek et al. 2011), the VCF file was filtered to remove indels and retain only biallelic sites with quality and depth of 30 and 5, respectively. Individual samples with more than 50% missing data were removed. One SNP per locus was randomly selected using the python script vcf_single_snp.py (Bongaerts 2018). Gene flow between clades was measured using ABBA‐BABA tests (Durand et al. 2011; Patterson et al. 2012) implemented using Dtrios in Dsuite v0.5 r53 (Malinsky et al. 2021) with the default number of Jackknife blocks.

*Morphological and taxonomic abbreviations* (also see Bond 2012)

**ANTd:** number of teeth on the anterior margin of the cheliceral fang furrow.

**Cl, Cw:** carapace length/width.

**AME, ALE, PME, PLE:** anterior median, anterior lateral, posterior median, and posterior lateral eyes.

**LBl, LBw:** labium length/width.

**PTl, PTw:** male palpal tibia length/width.

**Bl:** palpal bulb length from embolus tip to the bulb base.

**PTLs, TSIII:** number of female prolateral patella/tibial spines leg III.

**STRl, STRw:** sternum length/width.

**FI:** length of femur, legI.

**PI:** length of patella, legI.

**TibI:** length of tibia, legI.

**MI:** length of metatarsus, legI.

**TarI:** length of tarsus, legI.

**FIV:** length of femur, legIV.

**PIV:** length of patella, legIV.

**TibIV:** length of tibia, legIV.

**MIV:** length of metatarsus, legIV.

**TarIV:** length of tarsus, legIV.

**PLS:** posterior lateral spinneret.

**TSp, TSr, TSrd:** number of male tibial spines in the prolateral, retrolateral, and retrolateral‐distal positions.

**ITC:** inferior tarsal claw.

BME (Bohart Museum of Entomology; Davis, California).

### 2.5 Morphological Analyses

We examined 16 continuous somatic characters for 84 adult females and 38 males, with an additional two secondary sexual characters in males adapted from Bond (2012) (S2). Measurements were taken using a Leica M165C stereomicroscope. Before analysis, measurements were standardized by individual carapace length and visually confirmed to be normally distributed using Q‐Q plots in the R base package (R Core Team).

One‐way analysis of variance (ANOVA) was performed for the three species hypothesis (see Results) where the character measurement was the response variable and clade was the categorical factor. If ANOVA results were significant (*p* < 0.05), Tukey's Honestly Significant Difference (HSD) post hoc test and compact letter display were performed using the multcomp v1.4.25 package (Piepho 2004).

Principal component analyses (PCA) were performed without standardization by carapace length and visualized using the ggplot2 v3.5.0 package (Wickham 2016) in R (R Core Team) following Hamilton et al. (2016) with ellipses representing the multivariate *t*‐distribution at the 95% confidence interval for each group.

### 2.6 Measurement, Characterization, and Illustration of Morphological Features for Taxonomic Description

Description of morphological features follows Bond (2012). Unique voucher numbers were assigned to all specimens (alphanumeric designations beginning with AP, BME, or MY); these data were added to each vial and can be used to cross‐reference all images, measurements, and locality data. All measurements are given in millimeters and made with a Leica MC205 dissecting microscope with the Leica Analysis Suite Software. Lengths of leg articles were taken sensu Bond 2012, figures 11–16. Leg I and Leg IV article measurements are listed in the species description as: femur, patella, tibia, metatarsus, tarsus. Carapace and leg coloration are described semi‐quantitatively using Munsell Color Charts (Windsor, NY) and are given as hue value/chroma.

Digital images of specimens were made using a BKPlus Digital Imaging System (Dun Inc. TM, Richmond, VA) recorded at multiple focal planes and then assembled into a single focused image using the computer program Helicon Focus (Helicon Soft Ltd., Ukraine).

Latitude and longitude for the collecting locality were recorded in the field using a Garmin Global Positioning System receiver (Garmin International Ltd., Olathe, KS) using WGS84 map datum.

## 3 Results

### 3.1 Data Summary

A total of 39 *Aptostichus simus* individuals and two congeneric outgroup individuals were processed for sequencing. Detailed information regarding sequencing results, assemblies, scaffold match, and alignment is available in DRYAD (Jochim et al. 2025: https://doi.org/10.5061/dryad.9p8cz8wt9). Average UCE read count per individual was 4,192,746 and ranged from 1,092,845 to 14,639,635. When all 41 individuals were analyzed, the average number of probe matched scaffolds per individual was 1119 and ranged from 517 to 1360. When mapping and correction workflows were performed on the smaller ingroup dataset (excluding individuals from Baja (S3 for preliminary phylogeny)), the average number of probe matched scaffolds was 1264 and ranged from 1024 to 1351. Locus occupancy minimums of 75%, 85%, and 95% resulted in locus counts of 1263, 995, and 352, respectively. The two Baja California specimens were collected in 1992 and preserved in 70% ethanol and yielded only degraded DNA. Thus, only the corrected dataset excluding individuals from Baja was used in downstream phylogenetic analyses.

### 3.2 Phylogenetic Analysis

**FIGURE 1:**
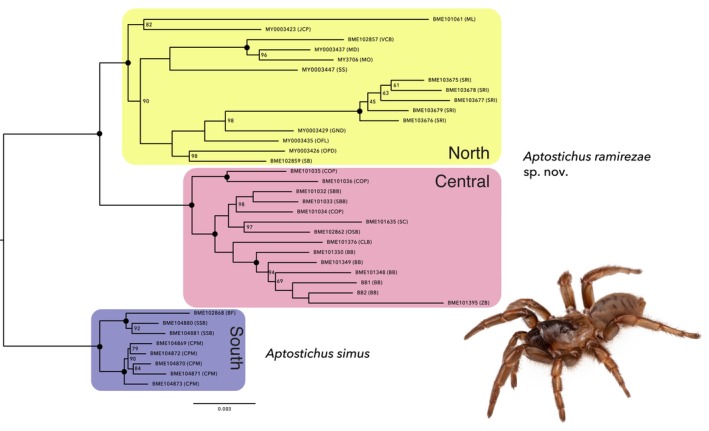
Maximum likelihood phylogram based on minimum 75% locus occupancy concatenated dataset. Shaded boxes correspond to major phylogenetic clades. Bootstrap support values < 100% are labeled, otherwise node support is 100%. Black circles indicate nodes with 100% support from consensus species trees generated with ASTRAL‐III with bootstrap resampling run with 100 pseudoreplicates in ASTRAL v5.7.8 Outgroup not shown for display purposes. Acronyms in parentheses next to specimen codes correspond to localities (see Figures 2 and S1). Subset image shows live *Aptostichus ramirezae* sp. nov. (BME101034).

**FIGURE 2:**
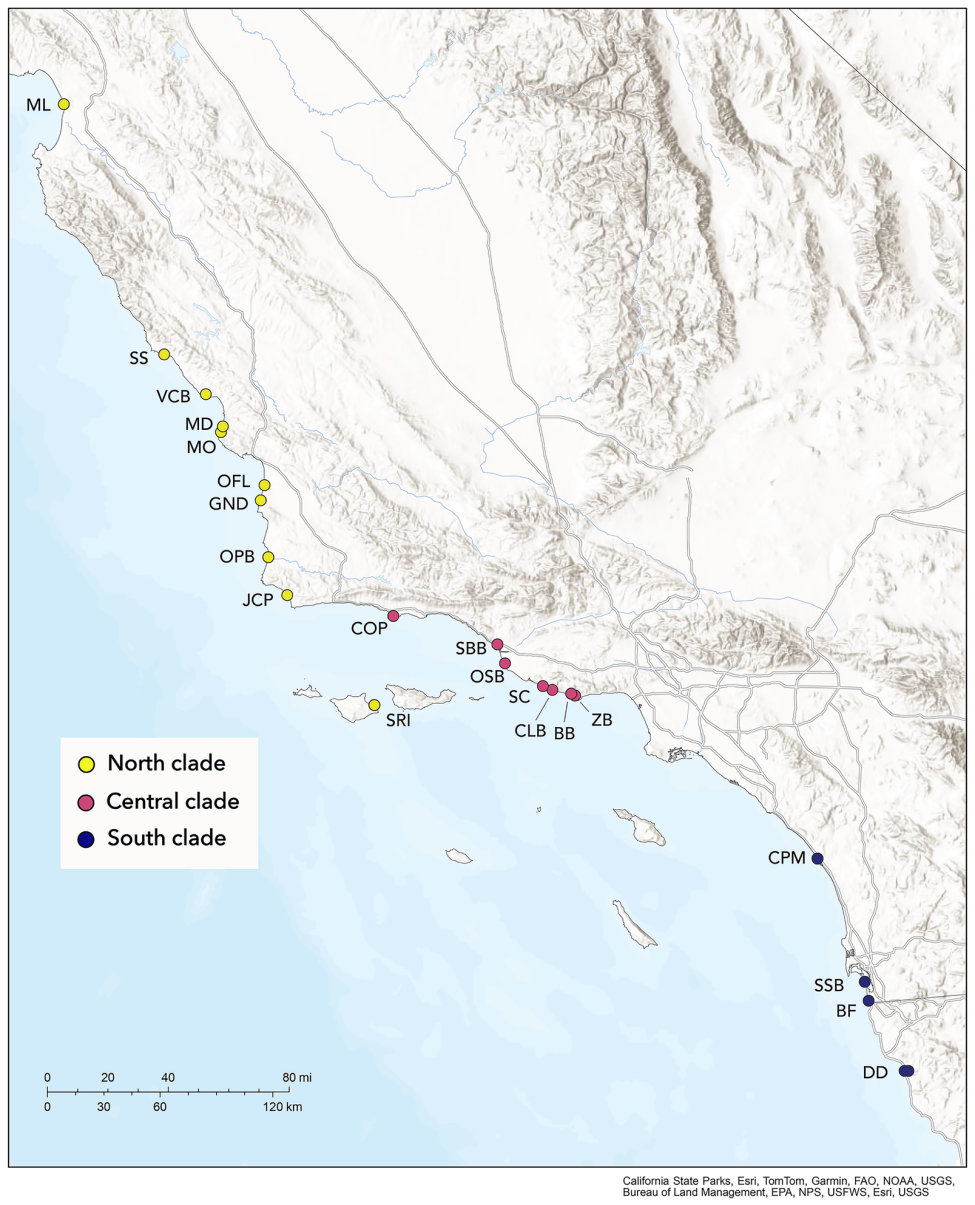
Distribution map of California and Baja California (partial) with localities (and their acronyms) included in genetic analyses. Colors correspond to major phylogenetic clades (see Figure 1). Map made with ArcGIS Online (Esri 2025).

Phylogenetic trees generated using maximum likelihood inference from concatenated UCE datasets with 75%, 85%, and 95% locus occupancy inferred the same three major clades with 100% support (Figures 1 and S4). We refer to these groups as the North, Central, and South clades based on geographic placement along the California coast (Figure 2). All consensus trees from the multispecies coalescent bootstrapping analysis recovered the same three major clades with 100% support (S5). Relationships within these clades are similar to those in the maximum likelihood phylogeny. Within the South clade, individuals from Silver Strand State Beach (SSB) and Border Field State Park (BF) group together and are sister to individuals from Camp Pendleton Marine Base (CPM). Within the Central clade, Coal Oil Point (COP) individuals are sister to the remaining Central clade individuals. Relationships within the North clade are not as resolved. In the coalescence‐based trees, individuals from Santa Rosa Island (SRI) are sister to the rest of North clade individuals, but in the maximum likelihood phylogeny, they are in a more derived position within the North clade, sister to the individual from Guadalupe‐Nipomo Dunes (GND).

### 3.3 SNP and Population Analyses

Three datasets, RANDSNP1, RANDSNP2, and RANDSNP3, each containing 806 unlinked SNPs for 32 ingroup individuals, were generated to visualize lineage clustering and estimate genetic differentiation between the major clades. Nucleotide diversity (π) estimates for the North, Central, and South clades were 0.16, 0.12, and 0.06, respectively. Percentages of missing data were 26%, 27%, and 27%, respectively. Global G'_ST_ was estimated at 0.68, with pairwise G'_ST_ values of 0.53 between North and Central, 0.71 between North and South, and 0.77 between South and Central. Jost's D was estimated at 0.20, with pairwise values of 0.13 between North and Central, 0.21 between North and South, and 0.24 between South and Central. Values for G'_ST_ and Jost's D were nearly identical across all SNP datasets; only RANDSNP1 is reported here.

**FIGURE 3:**
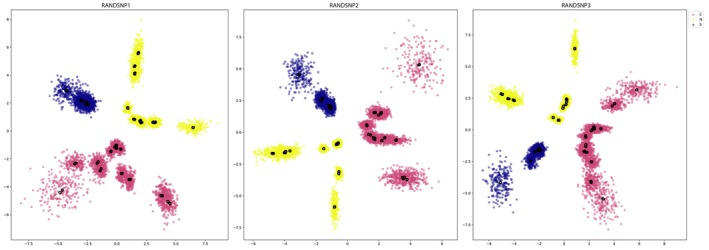
Variational AutoEncoder cluster analyses of three random unlinked SNP datasets. All datasets are based on the minimum 75% locus occupancy. Black open circles represent the mean of phased SNP data for an individual and color clouds represent the standard deviation for the latent distribution. Colors are based on assignment to the major clades (see Figure 1).

VAE analyses for the three SNP datasets revealed complete separation of the North, Central, and South clades. Visualization of VAE analyses indicates that the South clade comprises one or two genetic clusters, the Central clade comprises two to five clusters, and the North clade comprises three to five clusters (Figure 3).

**FIGURE 4:**
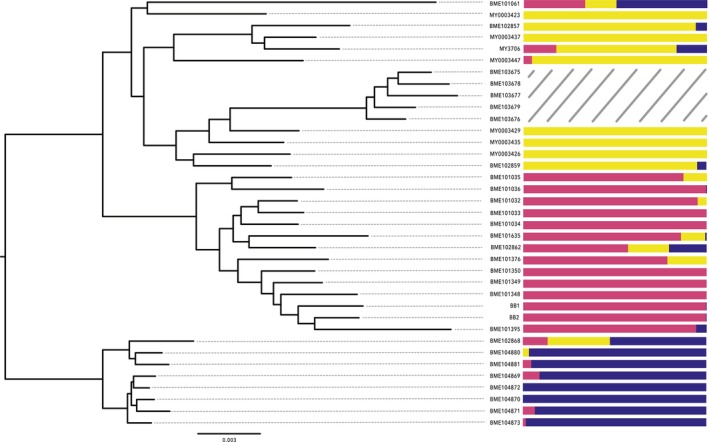
Results of maximum likelihood phylogeny with 75% locus occupancy (see S4a for exact bootstrap values). Ancestry proportions based on RANDSNP1 dataset generated using sNMF are plotted next to individuals. Individuals from Santa Rosa Island (indicated by hatch marks) were not included in this analysis due to higher levels of divergence and missing data.

Genetic clustering with sNMF recovered minimum cross‐entropy values when *K* = 3 for RANDSNP1 and RANDSNP3 datasets and *K* = 4 for the RANDSNP2 dataset (S6). This indicates that three ancestral populations optimally predict the genotypes of individuals in these analyses. A *K* value of 3 was used to plot ancestry proportions (Figures 4 and S7).

After filtering and SNP calling, the VCF file contained 846 randomly chosen unlinked biallelic SNPs for 37 ingroup individuals. ABBA‐BABA tests estimated Patterson's D statistic at 0.061 with a *p*‐value of 0.405 when North, Central, and South clades were assigned to P1, P2, and P3, respectively. The admixture fraction, or f_4_‐ratio, is 0.019.

### 3.4 Morphological Analysis

Males: Results of ANOVA showed that nine of the 17 measurements standardized by carapace length were significantly different between at least two clades (Table 1). Tukey's HSD test indicated that for PTl there is a significant difference in means between South‐Central (*p* < 0.0001) and South–North (*p* < 0.0001), but not between Central‐North (*p* = 0.97). For PTw there is a significant difference in means between South‐Central (*p* < 0.05) and South–North (*p* < 0.05), but not between Central‐North (*p* = 0.96). For TibI there is a significant difference in means between South‐Central (*p* < 0.0001) and South–North (*p* < 0.0001), but not between Central‐North (*p* = 0.79). For MI there is a significant difference in means between South‐Central (*p* < 0.01) and South–North (*p* < 0.0001), but not between Central‐North (*p* = 0.83). For STRl there is a significant difference in means between South‐Central (*p* < 0.05), but not South–North (*p* = 0.76) or Central‐North (*p* = 0.07). There is a significant difference in means for FI between South–North (*p* < 0.001), but not South‐Central (*p* = 0.09) or Central‐North (*p* = 0.68). For PI there is a significant difference in means between South–North (*p* < 0.001), but not South‐Central (*p* = 0.2) or Central‐North (*p* = 0.42). For PIV there is a significant difference in means between South–North (*p* < 0.05), but not South‐Central (*p* = 0.89) or Central‐North (*p* = 0.26). For MIV there is a significant difference in means between South–North (*p* < 0.01), but not South‐Central (*p* = 0.11) or Central‐North (*p* = 0.91).

**TABLE 1:** Results of ANOVA of morphological characters for 38 adult males.

Character	*F*	Pr(>*F*)	North	Central	South
**Palpal tibia length**	22.6	4.99e‐07***	a	a	b
**Palpal tibia width**	6.016	0.00568**	a	a	b
Carapace width	3.125	0.0564	—	—	—
Sternum length	4.311	0.0212*	ab	a	b
Sternum width	0.907	0.413	—	—	—
Labium length	2.551	0.0924	—	—	—
Labium width	1.763	0.186	—	—	—
Femur I length	8.589	0.000923***	a	ab	b
Patella I length	8.341	0.00109**	a	ab	b
Tibia I length	28.87	3.93e‐08***	a	a	b
Metatarsus I length	15.39	1.6e‐05***	a	a	b
Tarsus I length	1.516	0.234	—	—	—
Femur IV length	1.224	0.306	—	—	—
Patella IV length	4.06	0.026*	a	ab	b
Tibia IV length	2.636	0.0858	—	—	—
Metatarsus IV length	5.919	0.00611**	a	ab	b
Tarsus IV length	1.506	0.236	—	—	—

*Note:* Measurements for all characters were standardized by carapace length. *F* and *p* value results are shown. *F* value is calculated as the ratio of between‐group variance to within‐group variance. *p* value represents the probability that a difference between group means (*F* value) is observed by chance. Asterisks in column 3 represent the level of significance as determined by ANOVA. Letters in North, Central, and South columns represent group assignments from Tukey HSD post hoc tests for significant results. Bolded characters represent secondary sexual characters, and nonbolded are somatic characters. See Methods for character definitions.

Females: Results of ANOVA showed that eight of the 15 measurements standardized by carapace length were significantly different between at least two clades (Table 2). Tukey's HSD test indicated that there is a significant difference in means for STRw between South‐Central (*p* < 0.05) and South–North (*p* < 0.0001), but not between Central‐North (*p* = 0.08). For FI, there is a significant difference in means between South‐Central (*p* < 0.05) and South–North (*p* < 0.001), but not between Central‐North (*p* = 0.67). For PI, there is a significant difference in means between South‐Central (*p* < 0.01) and South–North (*p* < 0.0001), but not between Central‐North (*p* = 0.07). For TibI, there is a significant difference in means between South‐Central (*p* < 0.0001) and South–North (*p* < 0.0001), but not between Central‐North (*p* = 0.59). For FIV, there is a difference in means between South‐Central (*p* < 0.01) and South–North (*p* < 0.05), but not between Central‐North (*p* = 0.5). For LBw, there is a difference in means between South–North (*p* < 0.001), but not South‐Central (*p* = 0.11) or Central‐North (*p* = 0.17). For PIV, there is a difference in means between South–North (*p* < 0.01), but not South‐Central (*p* = 0.19) or Central‐North (*p* = 0.42). For TarIV, there is a difference in means between South–North (*p* < 0.05), but not South‐Central (*p* = 0.48) or Central‐North (*p* = 0.48).

**TABLE 2:** Results of ANOVA of morphological characters for 84 adult females.

Character	*F*	Pr(>*F*)	North	Central	South
Carapace width	1.475	0.235	—	—	—
Sternum length	2.665	0.0757	—	—	—
Sternum width	13.11	1.17e‐05***	a	a	b
Labium length	0.594	0.554	—	—	—
Labium width	8.681	0.000384***	a	ab	b
Femur I length	7.98	0.000686***	a	a	b
Patella I length	16.97	6.98e‐07***	a	a	b
Tibia I length	24.01	6.47e‐09***	a	a	b
Metatarsus I length	0.54	0.585	—	—	—
Tarsus I length	1.579	0.212	—	—	—
Femur IV length	5.994	0.00374**	a	a	b
Patella IV length	5.348	0.00658**	a	ab	b
Tibia IV length	0.151	0.86	—	—	—
Metatarsus IV length	1.704	0.188	—	—	—
Tarsus IV length	3.114	0.0498*	a	ab	b

*Note:* Measurements for all characters were standardized by carapace length. *F* and *p* value results are shown. *F* value is calculated as the ratio of between‐group variance to within‐group variance. *p* value represents the probability that a difference between group means (*F* value) is observed by chance. Asterisks in column 3 represent the level of significance as determined by ANOVA. Letters in the North, Central, and South columns represent group assignments from Tukey HSD post hoc tests for significant results. See Methods for character definitions.

**FIGURE 5:**
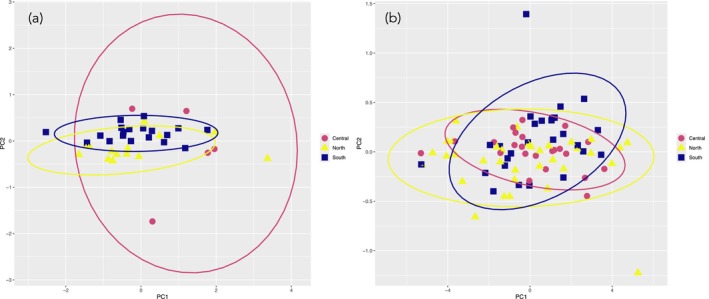
Visualization of principal components 1 and 2 for morphological measurements. (a) males (b) females. Ellipses are based on the multivariate *t‐*distribution at the 95% confidence level.

For the PCA of male continuous morphological traits, PC1, PC2, and PC3 accounted for 71%, 8%, and 7% of the total variation, respectively (S8). Although all ellipses overlap, the ellipse for the Central clade is much larger than those for the North and South clades, indicating variation among individuals and/or uncertainty due to a smaller sample size (Figure 5a). For the PCA of female continuous morphological traits, PC1, PC2, and PC3 accounted for 91%, 2%, and 2% of the total variation, respectively (S9). All ellipses highly overlap (Figure 5b).

## 4 Discussion

The phylogeny recovered three major clades in *Aptostichus simus:* the North, Central, and South clades. The South clade, populations in San Diego County and Baja Norte, is the earliest diverging. This result is consistent with Bond et al. (2001) which recovered a monophyletic grouping of haplotypes from the San Diego area. Additionally, both studies find that populations on the LA Basin coast, the Central clade, group phylogenetically. However, Bond et al. (2001) recovered the Santa Rosa Island population as sister to the LA Basin, whereas the present study indicates that it is nested within the North clade. This discrepancy could be the result of low sample sizes for northern localities in Bond et al. (2001) as they only included two localities from Monterey County, whereas this study includes an additional nine localities between Monterey and LA Basin populations. These minor discrepancies aside, the sub‐genomic data do indeed support a multi‐species hypothesis; that is, *A. simus* is “undersplit.”

### 4.1 Integrative Species Delimitation

Our preferred framework is an integrative method of reciprocal illumination in which we first test for monophyly, identify candidate species, and then assess for potential secondary defining properties. Integrative taxonomic methods have been used to uncover cryptic species in a diversity of vertebrate and invertebrate taxa (Blasco‐Costa et al. 2010; Bond and Stockman 2008; Derkarabetian and Hedin 2014; Funk et al. 2012; Padial and De La Riva 2009; Steiner et al. 2018; Weston et al. 2020). The species concept we employ is the Cohesion Species Concept (CSC; Templeton 1989), which defines species in terms of their genetic and phenotypic cohesion. Under the CSC, lineages are a species if they (1) are derived from a single evolutionary lineage and (2) are genetically and/or ecologically exchangeable (see Bond and Stockman 2008). The first requirement is fulfilled if the lineages of interest form a monophyletic group (genetic exclusivity). Our sub‐genomic phylogeny recovered three monophyletic groups that exhibit deep molecular divergence indicated previously by mitochondrial data (Bond et al. 2001). Although genetic exclusivity illuminates “candidate species,” such genetic divergence is not a taxonomic character (Wüster et al. 2024). Taxonomy is a hypothesis‐driven field of study; thus, it is crucial to state falsifiable hypotheses regarding species boundaries prior to claiming new species (Bond et al. 2021). Because diagnostic features, secondary defining properties, are the basis upon which new, nominal species are described, we then tested these species hypotheses using additional analyses of the genetic data and morphologic characters.

Species validation steps consisted of the concomitant analysis of genetic and morphological data. We first employed an unsupervised machine learning approach to visualize clusters in our SNP data. VAE analyses conducted on all three SNP datasets showed complete separation of the three major clades (Figure 3). We also employed sNMF to identify clusters in our data. These analyses indicated that genetic contributions from three ancestral populations optimally predicted masked individual genotypes (S6). Ancestry proportions for the 32 individuals included in the analysis indicated that the majority of genetic contributions came from the same clades recovered in our phylogenetic analysis (Figure 4). One exception is BME101061, an individual who is in the North clade but has larger ancestry proportions from the South and Central clades. This individual was collected from the northernmost locality, which is separated from the nearest population by approximately 70 miles of rocky coastline, making gene flow between BME101061 and the individuals from the South or Central clades extremely unlikely. This anomaly may be due to incomplete lineage sorting but is likely explained by the relatively high amount of missing SNP calls (56%) for this specimen.

The SNP data were also used to estimate genetic differentiation between the three clades. Global G'_ST_ for the three species hypothesis is 0.68. Pairwise G'_ST_ values are 0.53 between North–Central, 0.71 between North–South, and 0.77 between Central–South, inferring higher genetic differentiation between the South and the other two clades than across *A. simus* as a whole. Pairwise Jost's D values support this with estimates of 0.13 between North–Central, 0.21 between North–South, and 0.24 between Central–South. Genetic differentiation between the North and Central clades is lower, and these metrics indicate possible gene flow between the two (Jost 2008; Meirmans and Hedrick 2010).

To identify potential introgression, we conducted an ABBA‐BABA test, which assumes that under a scenario of incomplete lineage sorting, it is equally likely that the North or Central clade individuals share alleles with the South clade. If this assumption is violated and there are significantly more similarities between the South and North clade or South and Central clade, we could infer recent gene flow between the pair with more similarities (Malinsky et al. 2021). Results of the ABBA‐BABA test did not indicate gene flow between the North and South or Central and South clades. These tests cannot compare sister species, so we do not have a D statistic relating to introgression between the North and Central clades, but Hedrick's G'_ST_ infers that the two clades have less genetic differentiation (noted above).

We then investigated morphological features of both males and females. In mygalomorph spiders, the infraorder to which *A. simus* belongs, morphological characters used to distinguish species are primarily male secondary sexual characteristics, female internal reproductive features, and somatic characters including spination, size, and shape (Bond 2012; Goloboff 1995; Hamilton et al. 2016; De Montes Oca et al. 2016). Visualization of principal component analyses of two continuous male secondary sexual characters and 16 continuous somatic characters for both sexes revealed overlap of all three clades in both males and females (Figure 5). This result is unsurprising as mygalomorphs are morphologically conservative (Castalanelli et al. 2014; Hamilton et al. 2016; Leavitt et al. 2015; Newton et al. 2020).

Individual characters standardized by carapace length showed strong and statistically significant evidence of morphological differentiation of the South clade. ANOVA and Tukey's HSD indicated that of the 17 male characters measured, nine were significantly different between the South and North clades or the South and Central clades. None were significantly different between the North and Central clades. Notably, both secondary sexual characters examined were different between the South and the other two clades (Table 1). Taken together, these morphological differences do not support the *three* species hypothesis; we hypothesize that the North and Central clades may compose a single species.

ANOVA results for females indicated eight of the 15 characters examined were significantly different. Post hoc tests revealed that these characters are all different between the South and North clades, but only five were different between the South and Central clades. For the remaining three characters, the Central clade was not different from the South or North clades (Table 2). Again, these results are somewhat unsurprising given morphological homogeneity (Hamilton et al. 2016; Hendrixson and Bond 2007; Newton et al. 2020; Satler et al. 2013), particularly in adult females (Bond 2012; Coyle 1968).

Taken altogether, the integrative framework and process of reciprocal illumination show strong evidence for three well‐established and deeply divergent *A. simus* clades. By employing the CSC, we show that the South clade is neither genetically exchangeable nor potentially demographically interchangeable with the Central+North clade. Examination of multiple lines of sub‐genomic data leads us to reject the hypothesis that recent gene flow between the South and Central+North clade is occurring. Morphological analyses show differences between the South‐Central and South–North, but not between Central‐North. As such, our data support the original hypothesis by Bond et al. (2001) that *A. simus* is a cryptic species complex.

### 4.2 Cryptic Species

A common misconception is that cryptic species are the result of very recent speciation (Bickford et al. 2007). This is likely not the case for *A. simus* as divergence times for the South clade are quite old, 1.5–6.3 million years ago (Bond et al. 2001). So, what are other potential explanations as to why this group is so genetically divergent without accompanying morphological change? Extreme environmental conditions have been shown to promote morphological stasis in animal groups living in underwater karst (Lefébure et al. 2006) and deep‐sea environments (Vrijenhoek 2009). Although coastal dune systems do not experience such extremes, trapdoor spiders in these environments do undoubtedly contend with far more dynamic conditions than their inland relatives (wind and shifting sands, tidal inundations, changes in substrate consistency, etc.).

Nonvisual mating systems, ostensibly like those in trapdoor spiders, also lend to speciation without morphological divergence (Bickford et al. 2007; Jones 1997). Pheromone signals have been demonstrated to play a role in the identification of potential mates in Lepidoptera (Byers and Struble 1990; Kozlov et al. 1996), Amphipoda (Stanhope et al. 1992), and araneomorph spiders (Adams et al. 2021, 2024). In Araneae, chemical signals have long been known to play a role in courtship and mating (Platnick 1971), with multiple examples in mygalomorphs (Copperi et al. 2019; Coyle and Shear 1981; Frank et al. 2023). Because these spiders have poor eyesight and fossorial life histories, another characteristic associated with speciation in the absence of morphological change (Jörger and Schrödl 2013), nonvisual cues could be the primary signals used to find mates. Although courtship has not been explicitly studied in *Aptostichus* spp., personal observations of mate pairings in a laboratory setting revealed that male *A. stephencolberti* , another coastal dune endemic, could sense a conspecific female from contact with her silk alone.

Efforts to uncover cryptic species in taxa lacking distinguishing characters are increasing (Jörger and Schrödl 2013; Lukhtanov 2019; Struck et al. 2018). As has always been the case with taxonomy, there is not going to be a single set of characters that will work for all taxa. However, what should always be applied are approaches that consider multiple independent lines of evidence (Dayrat 2005). Accurately identifying cryptic diversity is necessary for gaining an understanding of Earth's biodiversity and has an important role in identifying areas of conservation concern (Bickford et al. 2007; Struck et al. 2018). In the case presented here, *A. simus* was previously considered a single widespread species endemic to threatened coastal dune habitats from Baja California Norte to Monterey County. In light of habitat fragmentation (Schlacher et al. 2014), human disturbance (Dugan and Hubbard 2010; Schlacher et al. 2014), and sea level rise impacting the California coast (Griggs 2021), the need to document and preserve remaining genetic diversity is critical for species and populations restricted to these habitats.

### 4.3 Coastal Dune Habitats and Conservation Implications

The coastal dune habitats in which *A. simus* are found are extremely dynamic environments (Sherman and Bauer 1993) and are of particular interest and conservation concern due to the variety of biotic and abiotic threats faced (McLachlan 1991; Schlacher et al. 2014). Trapdoor spiders can be found in almost all Californian coastal dune systems from Baja California Norte to Oregon (Bond 2012). Coastal dunes always have an environmental gradient perpendicular to the shore where fauna correspond to floral zones (McLachlan 1991). Araneomorph spiders (e.g., crab spiders, wolf spiders, etc.) can be found in all coastal dune zones from the drift‐line to dune meadows (Duffey 1968). Within California dunes, mature trapdoor spiders are found on and between the primary dune crest and secondary dune crest because vegetation structure and microclimate are presumably more stable there, and the risk of flooding from high tides is minimized (McLachlan 1991).

Despite the high diversity of fauna found in coastal dunes, most animal species found there are not true dune endemics. Because of the small area of most coastal dune fields and proximity to both land and sea, many taxa are able to move between zones and are thus not restricted to certain areas of the dunes (Duffey 1968; McLachlan 1991). Consequently, these trapdoor spiders are unique because they are found only in this habitat type. Due to their fossorial nature and low vagility, *A. simus* trapdoor spiders do not have the ability to quickly adapt to new zones, particularly within an individual's lifetime. This places dispersal‐limited taxa at high risk of extinction (Henle et al. 2004).

The trapdoor spider *Cryptocteniza kawtak* is one such example of a coastal dune species that was likely more widespread historically but is now found at a single locality (Bond et al. 2020). Although not coastal dune species, *Aptostichus killerdana* and *A. lucerne* are two more examples of trapdoor spiders with an extremely limited range that are now presumed extinct (Bond 2012). In our 2021 and 2022 collection efforts, we could not find any *A. simus* individuals from Broad Beach in Malibu, CA. Spiders were collected at this locality in the 1990s, but the habitat has experienced severe degradation due to human development.

In addition to human development (Harden 2004), habitat degradation (Dugan and Hubbard 2010; Hotten 1988), and coastal squeeze (Schlacher et al. 2014), dune endemics are at risk of extinction due to the effects of climate change, particularly rising sea level. The effects of rising sea level on the California coast can be observed in more extensive coastal flooding during storms and increased coastal erosion (Griggs 2021). Between the years 1900 and 2020, San Diego has experienced a mean sea‐level rise of 2.13 mm/year. Mean sea‐level rise for this area is projected to increase to 4.1–6.2 mm/year. by the year 2030 (Griggs 2021). This estimate is particularly concerning for the future of *A. simus* genetic diversity as the San Diego populations (the South clade) are more genetically distinct than the North and Central clades are to each other.

### 4.4 Taxonomic Treatment

Taxonomy, the science of documenting and describing Earth's biodiversity, is pivotal in any efforts to conserve said diversity. It is impossible to protect a species if it is unknown or undescribed as the majority of existing species are (Mora et al. 2011; Stork 2018). As discussed above, our phylogenetic analysis of UCE data delineated three major clades in what is currently described as *A. simus*. The integrative approach employed inferred strong evidence for at least one additional cryptic species that includes those populations comprising the North+Central clade (the South clade includes the type locality for *A. simus* at Silver Strand State Beach in San Diego Co.). Because we have rejected the CSC criteria of all clades composing a single genetically exchangeable and demographically interchangeable species coupled with dire conservation concerns, formally describing a new species is warranted.

## 5 Conclusions

An integrative framework for taxonomy is one that includes multiple lines of evidence (e.g., morphological, genetic, behavioral) analyzed with a variety of methods (Dayrat 2005). In the case presented here, there are no obvious morphological differences between the three major clades of *Aptostichus simus*. However, deep molecular divergence, which could be indicative of cryptic speciation, was detected in mitochondrial data over 20 years ago (Bond et al. 2001). These genetic data warranted a reexamination of species boundaries in this group with modern methods. Our chosen framework employed somatic and secondary sexual morphological characters and multiple genetic data types to uncover cryptic diversity in what was previously a single species. *Aptostichus simus* and *A. ramirezae* sp. nov. provide an interesting case study of the speciation continuum. In this group, we have closely related populations exhibiting different levels of reproductive isolation and deep molecular divergence between the two nominal species despite any obvious morphological changes. This study will inform future efforts to re‐evaluate species boundaries in taxa that may be harboring cryptic diversity.

**FIGURE 6:**
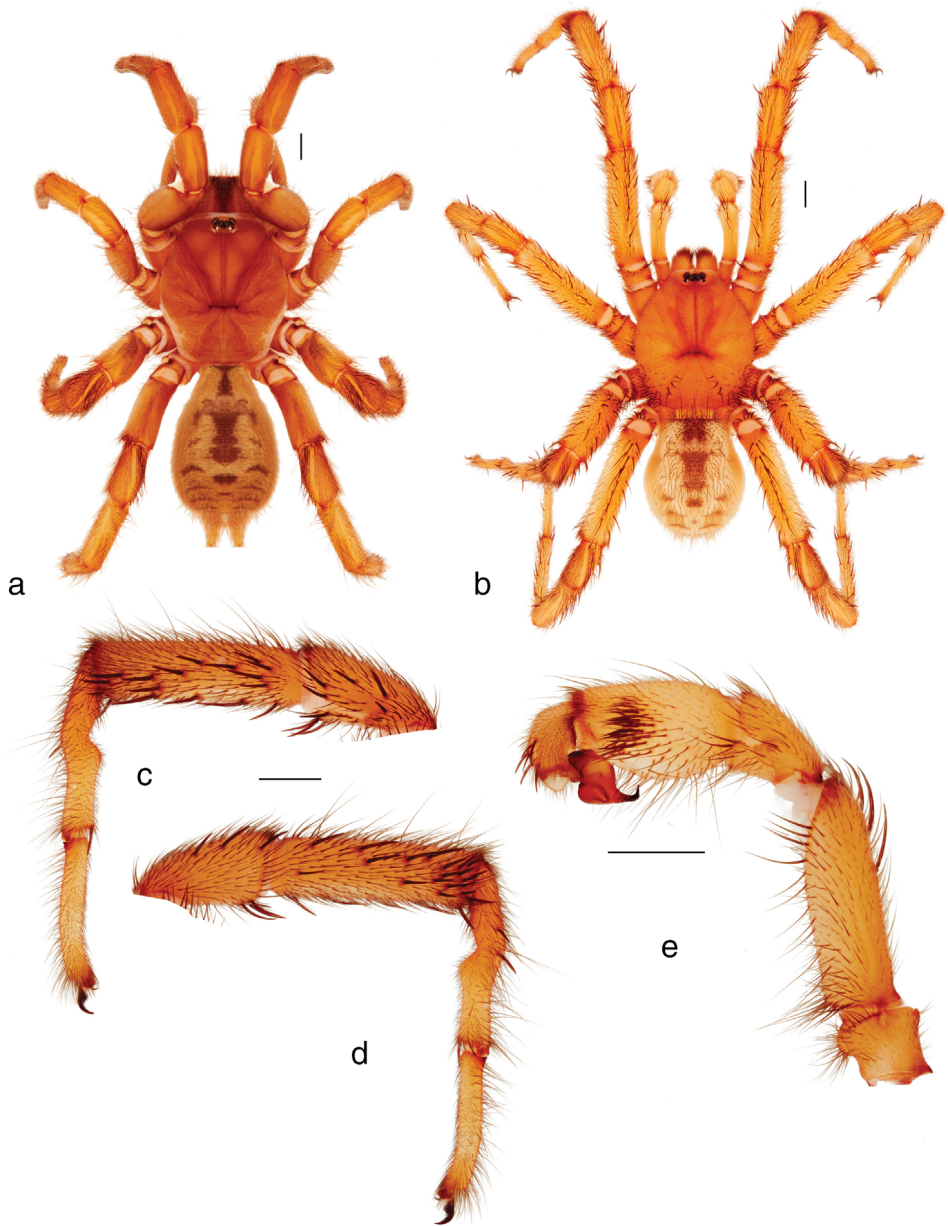
*Aptostichus ramirezae* sp. nov. (a) PARATYPE female (MY3749) habitus. (b–e) HOLOTYPE male (BME101067). (b) habitus, (c) retrolateral view of leg I, (d) prolateral view of leg I, (e) retrolateral view of pedipalp. Scale bars = 1 mm.

**Taxonomy** (Figure 6)

Family Euctenizidae Raven: 1985

*Aptostichus* Simon, 1891

*Aptostichus ramirezae* sp. nov. Jochim & Bond

urn:lsid:zoobank.org:pub:39480A99‐171B‐4638‐B5CC‐72925E5ACA1E

**Diagnosis**. *Aptostichus ramirezae* is very similar to *A. simus*, yet genetically divergent and can be diagnosed using a combination of quantitative characteristics. Males have a serrated embolus (Figure 6e) comprising 4–10 serrations, whereas the number of serrations in *A. simus* is on average lower (3–9). Male palpal tibia length and width are smaller in *A. ramirezae* (1.76 and 1.05 mm) when compared to overall body size. Females of both species have a very large patch of endite cuspules; *A. ramirezae* 304 ± 110. Female leg I femur, patella, and tibia are shorter in *A. ramirezae* (5.09, 3.27, and 3.45 mm, respectively) compared to *A. simus* (5.31, 3.49, and 3.76 mm, respectively). This species is restricted to coastal dune habitats in California, United States from Moss Landing State Beach, Monterey Co. to El Segundo Beach, Los Angeles Co. Disjunct populations are found on Santa Cruz Island and Santa Rosa Island.

**Etymology**. The specific epithet is a matronym in honor of Dr. Martina G. Ramirez whose work on *Aptostichus simus* provided many of the specimens used in our analyses. We admire her career dedication to supporting groups underrepresented in STEM fields; J.E.B. is particularly grateful to Dr. Ramirez for suggesting *Aptostichus* as a potential study group so many years ago and for her support through donation of specimens.

**Type material**. HOLOTYPE MALE (BME101067; deposited in the BME) from United States, California, Monterey Co., Moss Landing State Beach, N 36.81462 W −121.79050, coll. by J. Bond, 1.x.2019. PARATYPE FEMALE (MY3749; deposited in the BME) from United States, California, Monterey Co., Moss Landing State Beach, N 36.80915 W −121.78831, coll. by J. Bond, W.A. Bond, 27.vii.2008.

**MALE HOLOTYPE**. *Specimen preparation and condition*. Specimen preserved in 80% EtOH. Pedipalp, leg I, and leg IV removed, stored with the specimen. *General coloration in alcohol*. Carapace yellowish red 5YR 4/6; abdomen lighter with darker mottling (Figure 6b). *Cephalothorax*. Carapace 5.29 long, 4.56 wide, slightly setose. Fringe with heavy setae. Thoracic fovea groove deep, recurved. Eyes on slightly raised tubercle. AER procurved, PER recurved. AME ~¾ diameter PME. Sternum setose, STRl 3.12, STRw 2.59. Posterior sigilla moderately sized, widely separated oval in shape; anterior sigilla pair smaller, at margin. ANTd with 4 denticles. Palpal coxa and labium lack cuspules, LBw 0.53, LBl 0.33. Weak rastellum, comprises ~10 stout spines not on a tubercle. Abdomen. Moderately setose; apical segment of PLS short, domed. Legs. Leg I: 4.74, 2.51, 3.46, 2.99, 2.31; leg IV: 4.42, 2.23, 3.67, 4.00, 2.86. Tarsi I‐IV with trichobothria increasing in irregular rows. ITC small, strongly curved. Preening brush on leg II. Leg I spination pattern (Figure 6c,d): TSp 18, TSr 11, TSrd 5. Pedipalp. PTw 0.98, PTl 1.64, Bl 0.802. Embolus arises sharply from copulatory bulb, thin taper distally (Figure 6e).

Variation (*n* = 19). Cl 5.00–7.09, 5.69 ± 0.59; Cw 4.19–5.87, 4.76 ± 0.47; STRl 2.44–3.96, 2.99 ± 0.36; STRw 2.29–3.08, 260 ± 0.23; LBl 0.33–0.71, 0.53 ± 0.12; LBw 0.53–0.96, 0.77 ± 0.12; Leg I 14.68–20.07, 16.79 ± 1.52; Leg IV 15.66–22.25, 18.28 ± 1.97; TSp 11–27, 15.59 ± 3.69; TSr 6–17, 10.65 ± 3.05; TSrd 4–10, 6.53 ± 1.71.

Additional material examined. UNITED STATES: CALIFORNIA: **Monterey Co:** Moss Landing State Beach [N 36.81462 W −121.79050, BME101058–BME101066], 1.x.2019 (J. Bond), 9♂. **San Luis Obispo Co:** Baywood [N 35.30837 W −120.86835, AP_621], 26.xi.1977 (P.H. Sullivan), 1♂. **Santa Barbara Co:** North end Carpinteria State Beach [34.39309–119.52390, AP_659], 27.ix.1961 (W. Gertsch, W. Ivie), 1♂; Santa Cruz Island, Johnson's Lee Beach [N 33.96840 W −119.82910, AP_658, BME103984], 03.x.1987 (M.G. Ramirez), 2♂; Surf Beach [N 34.68263 W −120.60596, BME103608], 15.ix.2022 (E. Jochim, L. Chamberland, I. Quayle), 1♂. **Los Angeles Co:** Los Angeles, El Segundo Sand Dunes [N 33.93900 W −118.43900, AP_441, AP_442], 09.xii.1987 (J. George), 2♂; Playa Del Rey Beach, sand dunes between intersection of 66th and Pacific and Pacific and Argonaut St [N 33.95900 W −118.44910, AP_611], 06.xi.1982 (M.G. Ramirez, H.E. David), 1♂. **Ventura Co:** Oxnard State Beach [N 34.17683 W −119.23609, BME103610], 16.ix.2022 (E. Jochim, L. Chamberland, I. Quayle), 1♂.

**FEMALE PARATYPE**. *Specimen preparation and condition*. Specimen preserved like male holotype. Color. Carapace dark reddish brown 5YR 3/4; abdomen lighter with darker mottling (Figure 6a). Cephalothorax. Carapace 5.81 long, 5.04 wide, with a single line of setae from the anterior margin to thoracic fovea. Lacks fringe. Thoracic fovea groove deep and straight to slightly procurved. Low tubercle. PER recurved, AER procurved. AME approximately half the diameter of PME. Sternum moderately setose, STRl 3.19, STRw 2.91. Posterior sigilla moderately sized, widely separated oval in shape. ANTd with 4 teeth. Palpal coxae, numerous cuspules, clustered proximally; labium lacks cuspules, LBl 0.753, LBw 0.973. Weak rastellum, comprises ~20 stout spines not on a tubercle. Legs. Leg I: 4.35, 2.75, 3.07, 2.25, 1.29; leg IV: 4.08, 2.47, 3.16, 2.84, 1.79; legs and pedipalps highly setose. Moderately dense scopulae on tarsi I and II and metatarsi I, scopulae on the distal half of metatarsi II. Tarsus IV with trichobothria increasing in length distally. PTLs 16, TSIII 4. ITC small, sharply curved. Preening brush on leg IV. Anterior spermathecae with moderately sized base and branched stalk. Apical segment of PLS short, domed.

Variation (*n* = 58). Cl 4.66–10.28, 7.02 ± 1.24; Cw 3.66–8.78, 5.97 ± 1.15; STRl 2.56–5.40, 3.96 ± 0.69; STRw 2.17–4.85, 3.42 ± 0.60; LBw 0.75–1.62, 1.11 ± 0.20; LBl 0.51–1.30, 0.85 ± 0.18; Leg I 10.58–21.21, 16.02 ± 2.63; Leg IV 11.62–22.81, 16.84 ± 2.56; TSIII 2–9, 5.71 ± 1.49.

Additional material examined. UNITED STATES: CALIFORNIA: **Los Angeles Co:** Playa Del Rey Beach, sand dunes between intersection of 66th and Pacific and Pacific and Argonaut St [N 33.95900 W −118.44910, AP_078], 06.xi.1982 (M.G. Ramirez, H.E. David), 1♀; Malibu, Broad Beach [N 34.03385 W −118.85172, AP_622, AP_626], (M.G. Ramirez), 2♀; Malibu, Sycamore Cove Beach [N 34.06970 W −119.01220, BME101632, BME101636], 17.vii.1992 (M.G. Ramirez), 2♀. **Ventura Co:** McGrath State Beach [N 34.22640 W −119.26140, AP_062], 25.vi.1982 (M.G. Ramirez, H.E. David), 1♀; Point Mugu [N 34.08639 W −119.05562, AP_596], 16.vi.1979 (D. Boe), 1♀; Point Mugu State Park, Sycamore Cove Beach [N 34.07428 W −119.02066, AP_627, AP_629], (M.G. Ramirez), 2♀; San Buenaventura State Beach [N 34.26914 W −119.27978, BME101032, BME101033], 29.xii.2019 (J. Bond), 2♀; Oxnard State Beach Park [N 34.17720 W −119.23680, BME102862, BME102863, BME103613], 15.vii.2021 (E. Jochim, L. Chamberland), 2♀, 16.ix.2022 (E. Jochim, L. Chamberland, I. Quayle), 1♀. **Monterey Co:** Salinas River State Beach [N 36.78310 W −121.79440, AP_1264, AP_757], 06.v.1997 (J. Bond), 2♀; Moss Landing State Beach [N 36.80861 W −121.78833, AP_624, MY3081, MY3534], 14.v.1997 (J. Bond), 1♀, 17.iii.2005 (J. Bond, D. Beamer, A. Stockman), 1♀, 30.i.2006 (A. Stockman, P. Marek), 1♀. **San Luis Obispo Co:** Morro Bay, beach north of Morro Rock [N 35.37390 W −120.86132, AP_1208], 03.iv.2005 (M. Hedin, S. Foldi), 1♀; Morro Dunes, south end of Morro Bay [N 35.30583 W −120.87282, MY3437, MY3439], 06.xii.2005 (J. Bond), 2♀; Morro Bay, north end [N 35.37488 W −120.86317, MY3441], 06.xii.2005 (J. Bond), 1♀; isolated dune field just north of San Simeon Bay [N 35.64667 W −121.21106, MY3447], 06.xii.2005 (J. Bond), 1♀; Dunes near Villa Creek Beach in Cayucos [N 35.45800 W −120.96500, BME102858], 13.vii.2021 (E. Jochim, L. Chamberland), 1♀. **Santa Barbara Co:** Santa Rosa Island, dunes on Skunk Point [N 33.98210 W −119.97930, AP_050, AP_057a, AP_057b, AP_057c, AP_1261], 11.viii.1994 (M.G. Ramirez, H.E. David), 5♀; Santa Rosa Island, Southeast Anchorage [N 33.97870 W −120.00340, AP_083, AP_092], 09.viii.1994 (M.G. Ramirez, H.E. David), 2♀; Surf Beach near Lompoc‐Surf Train Station [N 34.68310 W −120.60570, BME102860, BME103606, BME103607], 14.vii.2021 (E. Jochim, L. Chamberland), 1♀, 15.ix.2022 (E. Jochim, L. Chamberland, I. Quayle), 2♀; Skunk Point on Santa Rosa Island [N 33.98100 W −119.99700, BME103674–BME103679], 08.x.2022 (E. Jochim, J. Starrett, W. Mendez), 6♀; Jalama County Park [N 34.50987 W −120.50115, MY3423], 17.iii.2005 (J. Bond, A. Stockman, D. Beamer), 6♀; Ocean Park Dunes [N 34.69005 W −120.60310, MY3425, MY3427], 17.iii.2005 (J. Bond, A. Stockman, D. Beamer), 2♀; Guadalupe‐Nipoma Dunes Preserve [N 34.96240 W −120.64973, MY3428, MY3429], 17.iii.2005 (J. Bond, A. Stockman, D. Beamer), 2♀; Oso Flaco Lake Preserve [N 35.03419 W −120.63260, MY3434, MY3435], 17.iii.2005 (J. Bond), 2♀; Coal Oil Point Reserve, U.C. Santa Barbara Reserve [N 34.40722 W −119.87720, AP_076, AP_623, AP_689, AP_697, AP_721, AP_743, BME101034, BME101035, BME103575], 24.vi.1982 (M.G. Ramirez, H.E. David), 1♀, 24.vii.1982 (M.G. Ramirez), 1♀, 21.xi.1998 (J. Bond), 2♀, 05.iv.1996 (J. Bond), 2♀, 29.xii.2019 (J. Bond), 2♀.

Distribution. California: Coastal dunes from Moss Landing State Beach, Monterey Co. to El Segundo Beach, Los Angeles Co. Disjunct populations found on Santa Cruz Island and Santa Rosa Island.

Natural history. Exclusively in coastal dune habitats. Males likely mature in early Autumn.

Conservation status. Using NatureServe (Master et al. 2012) Conservation Status Rank criteria, we consider the status of *Aptostichus ramirezae* to be CRITICALLY IMPERILED because of its high risk of extinction due to a very restricted range, extremely low probability of immigration due to fragmented habitat, population bias towards adult females and juveniles, and very narrow environmental specificity.

## Author Contributions

**Emma E. Jochim:** conceptualization (equal), data curation (equal), formal analysis (equal), funding acquisition (supporting), visualization (equal), writing – original draft (lead), writing – review and editing (lead). **James Starrett:** conceptualization (equal), data curation (equal), formal analysis (supporting), funding acquisition (supporting), investigation (supporting), writing – review and editing (supporting). **Hanna R. Briggs:** data curation (equal), writing – review and editing (supporting). **Jason E. Bond:** conceptualization (equal), funding acquisition (lead), resources (lead), supervision (supporting), visualization (supporting), writing – original draft (supporting), writing – review and editing (equal).

## Conflicts of Interest

The authors declare no conflicts of interest.

## Supporting information


**Appendix S1:** ece372346‐sup‐0001‐AppendixS1.tif.

**Appendix S2:** ece372346‐sup‐0002‐AppendixS2.pdf.

**Appendix S3:** ece372346‐sup‐0003‐AppendixS3.pdf.

**Appendix S4:** ece372346‐sup‐0004‐AppendixS4.pdf.

**Appendix S5:** ece372346‐sup‐0005‐AppendixS5.pdf.

**Appendix S6:** ece372346‐sup‐0006‐AppendixS6.pdf.

**Data S1:** ece372346‐sup‐0007‐DataS1.xlsx.

**Data S2:** ece372346‐sup‐0008‐DataS2.xlsx.

**Data S3:** ece372346‐sup‐0009‐DataS3.xlsx.

**Appendix S7:** ece372346‐sup‐0010‐AppendixS7.docx.

## Data Availability

Detailed information regarding sequencing results, assemblies, scaffold match, and alignment is provided in DRYAD (Jochim et al. 2025: https://doi.org/10.5061/dryad.9p8cz8wt9). Demultiplexed raw reads are available in the GenBank Sequence Read Archive database (PRJNA1216726). All scripts used in this study can be found here: https://github.com/emmajochim/AptSimus_SpDelim.
