# Supplementary figures and images for: Speciation Pattern and Process in the California Coastal Dune Endemic Trapdoor Spider Aptostichus simus (Mygalomorphae: Euctenizidae) and Description of a New Cryptic Species
Source: Ecol Evol. 2025 Oct 22;15(10):e72346. doi: 10.1002/ece3.72346 (PMC12541282; doi:10.1002/ece3.72346)

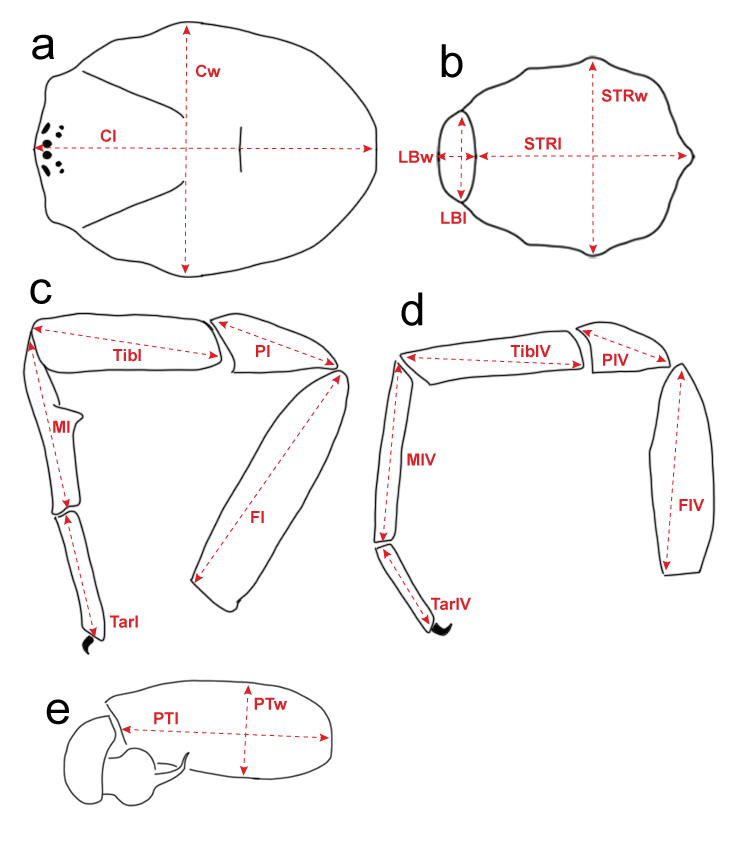

Supplement: Supplementary file 1 — Appendix S1: ece372346‐sup‐0001‐AppendixS1.tif. [file ECE3-15-e72346-s004.tif]

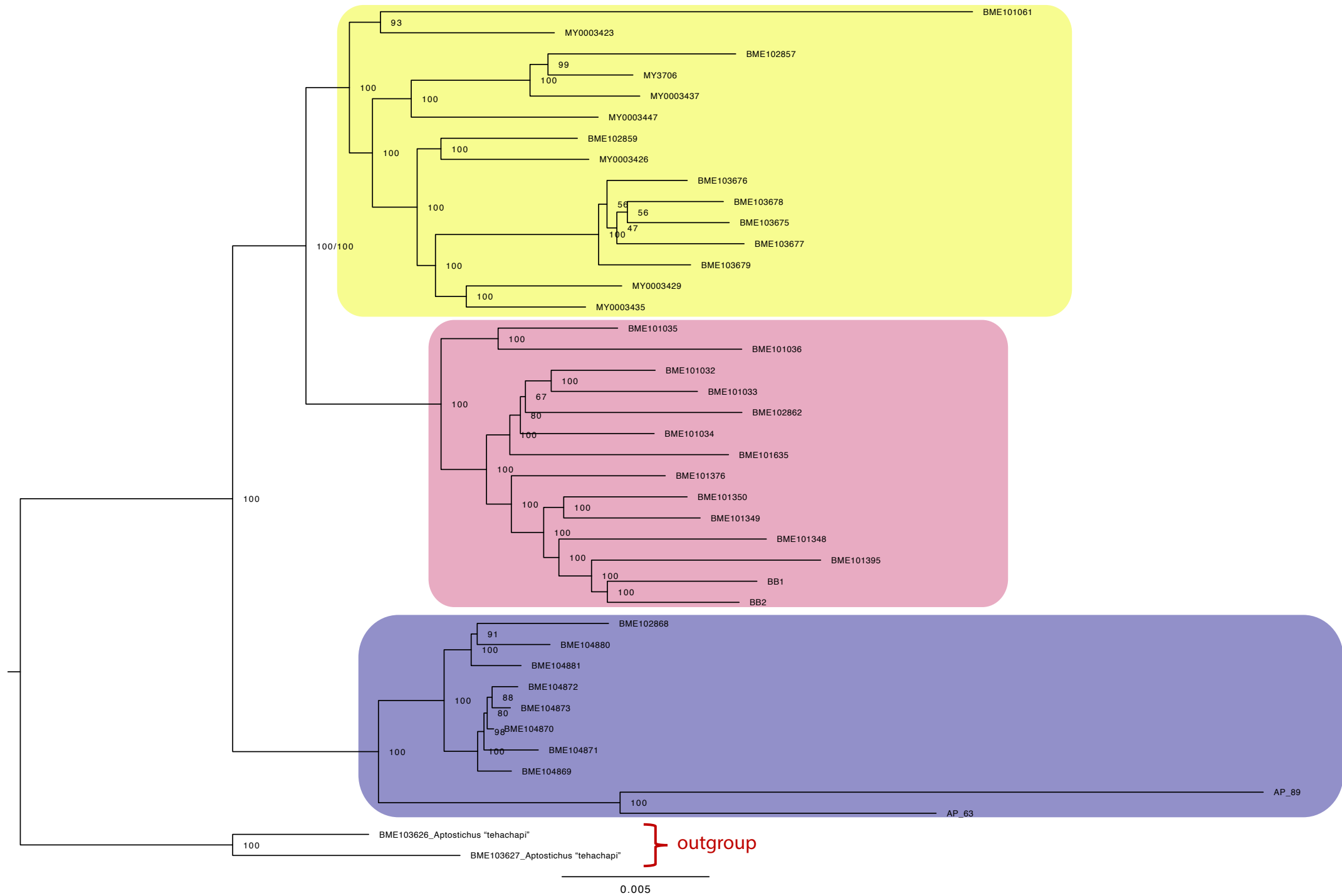

Supplement: Supplementary file 2 — Appendix S2: ece372346‐sup‐0002‐AppendixS2.pdf. [file ECE3-15-e72346-s001.pdf]

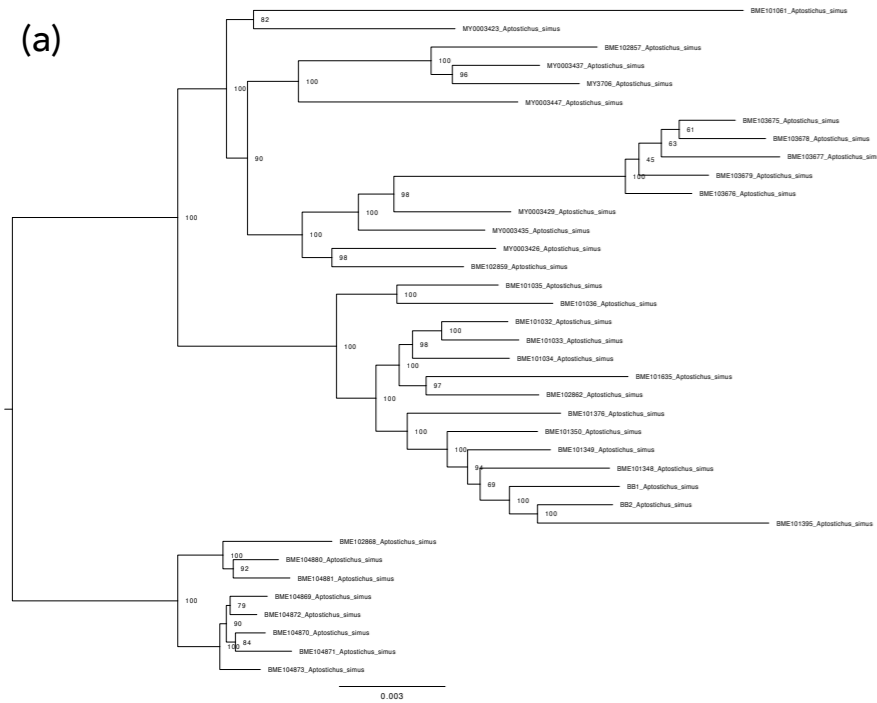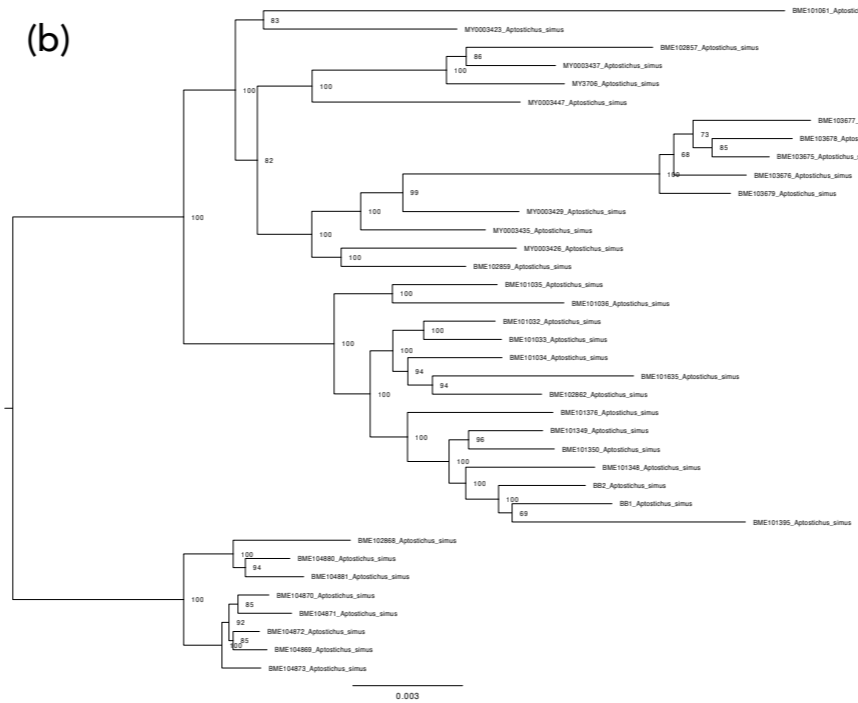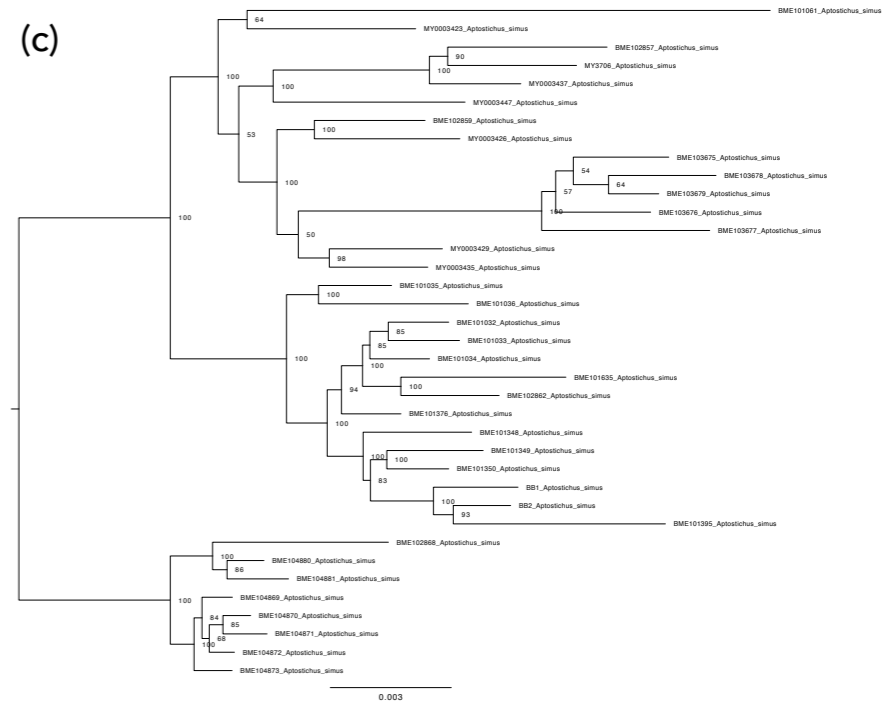

Supplement: Supplementary file 3 — Appendix S3: ece372346‐sup‐0003‐AppendixS3.pdf. [file ECE3-15-e72346-s006.pdf]

(a)

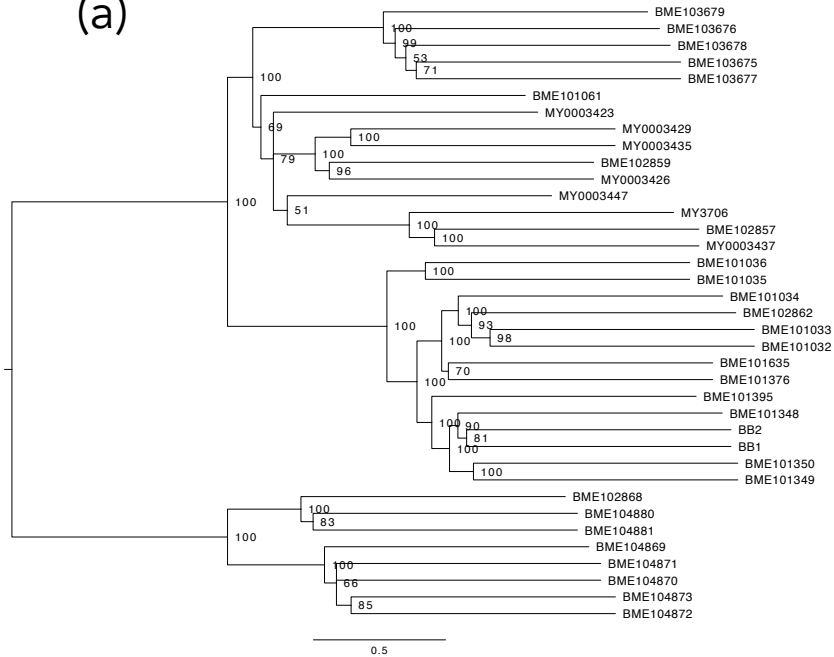

50% consensus

(b)

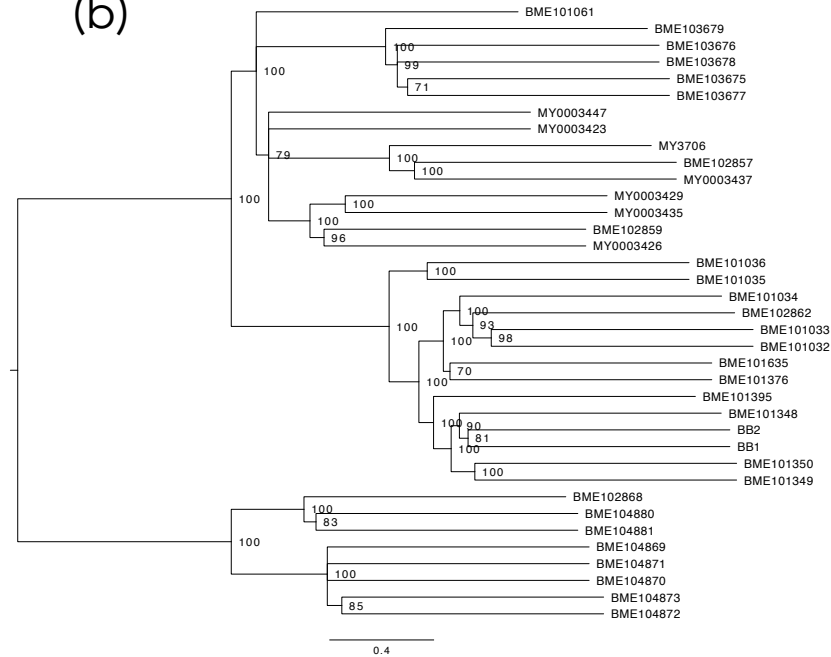

70% consensus

(c)

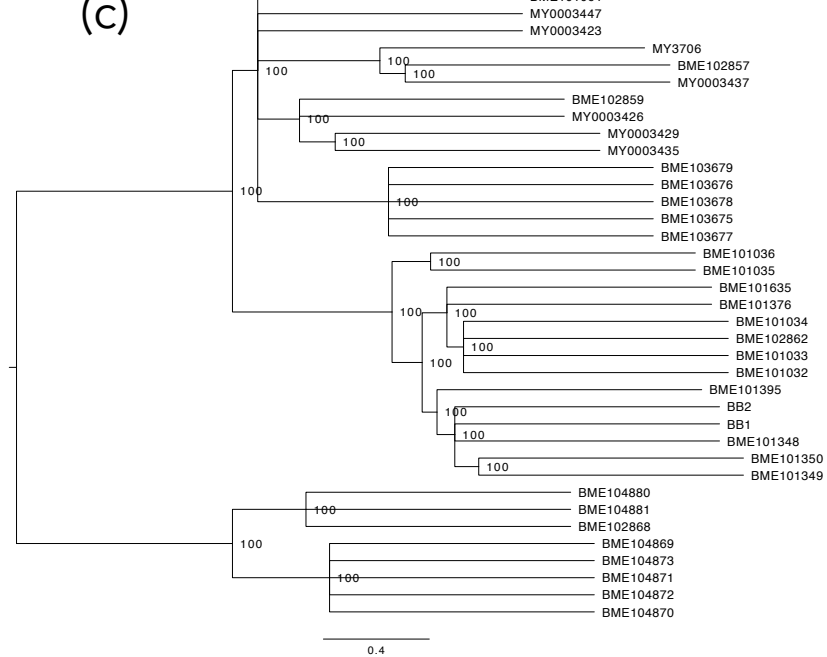

100% consensus

Supplement: Supplementary file 4 — Appendix S4: ece372346‐sup‐0004‐AppendixS4.pdf. [file ECE3-15-e72346-s003.pdf]

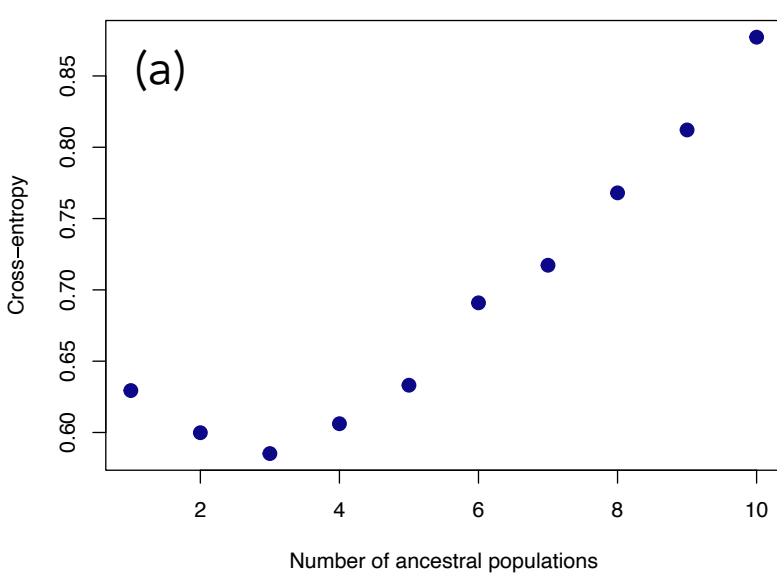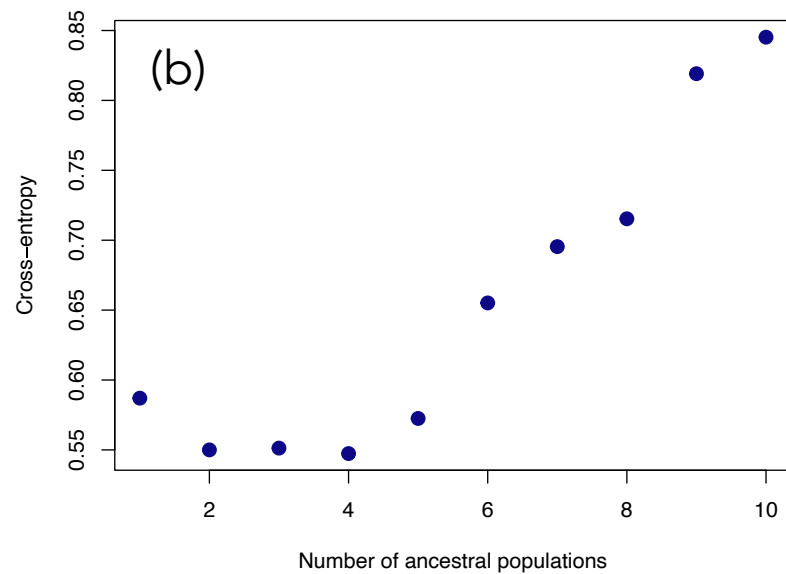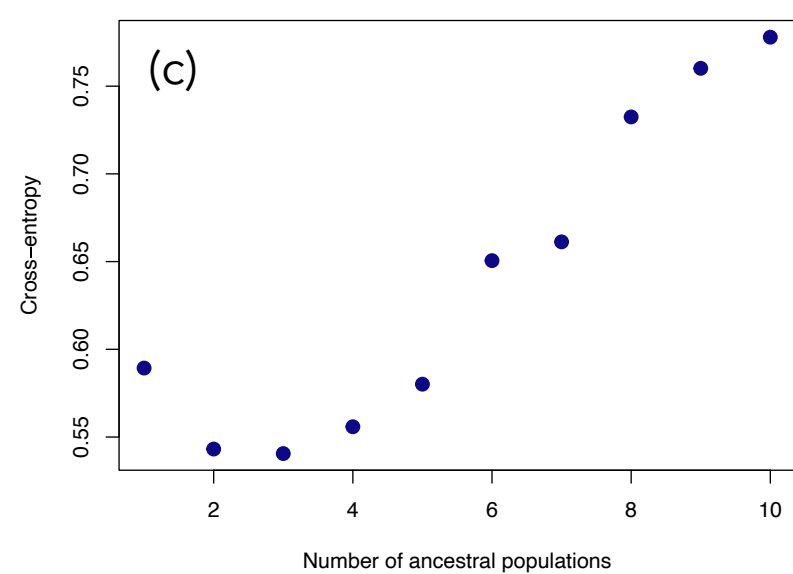

Supplement: Supplementary file 5 — Appendix S5: ece372346‐sup‐0005‐AppendixS5.pdf. [file ECE3-15-e72346-s008.pdf]

# RANDSNP2

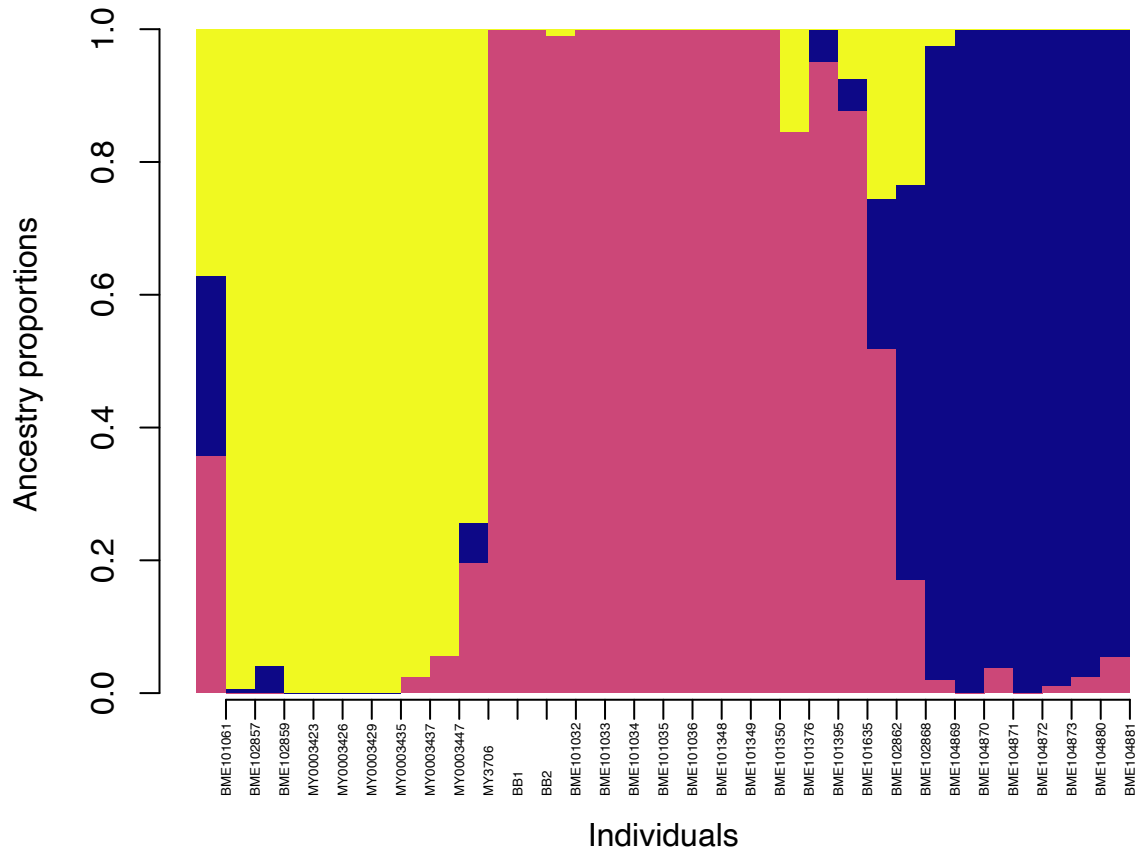

# RANDSNP3

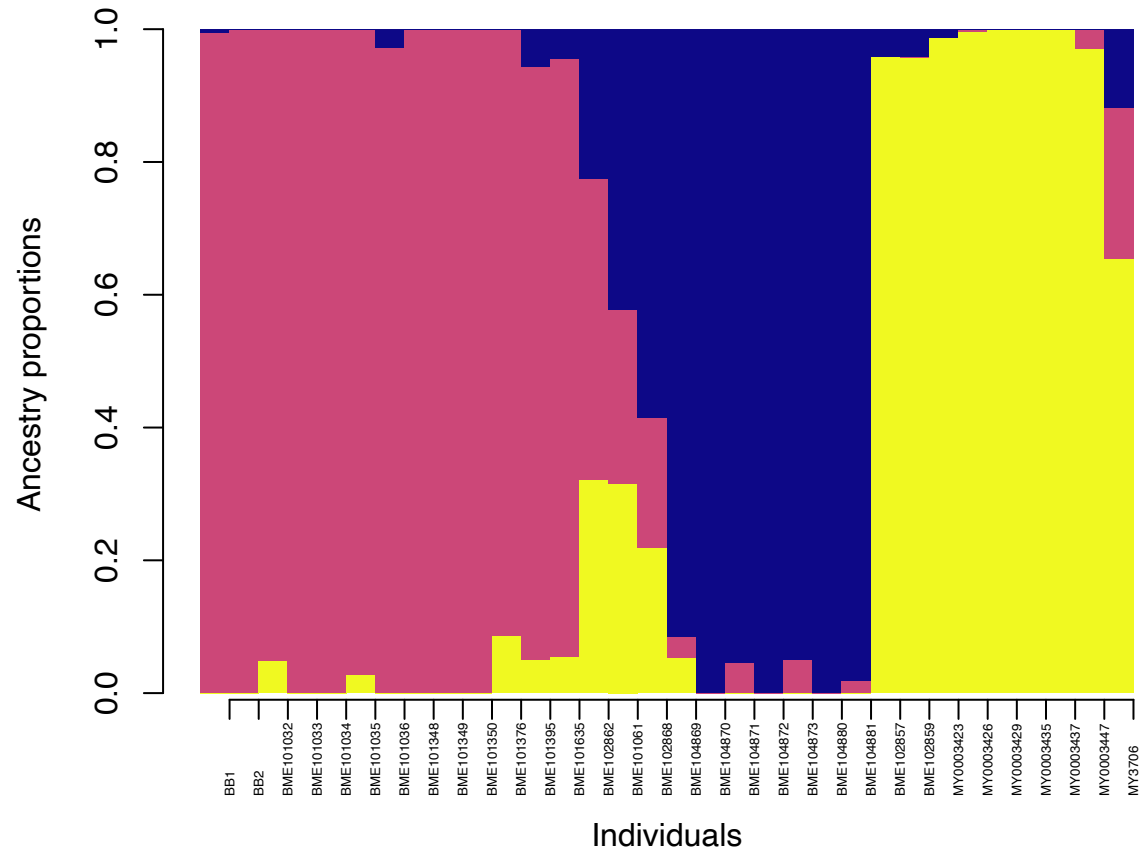

Supplement: Supplementary file 6 — Appendix S6: ece372346‐sup‐0006‐AppendixS6.pdf. [file ECE3-15-e72346-s002.pdf]
