# Supplementary material for: Speciation Pattern and Process in the California Coastal Dune Endemic Trapdoor Spider Aptostichus simus (Mygalomorphae: Euctenizidae) and Description of a New Cryptic Species
Source: Ecol Evol. 2025 Oct 22;15(10):e72346. doi: 10.1002/ece3.72346 (PMC12541282; doi:10.1002/ece3.72346)
Supplement: Supplementary file 10 — Appendix S7: ece372346‐sup‐0010‐AppendixS7.docx. [file ECE3-15-e72346-s009.docx]

**Supplemental Methods/Results—BPP**

We employed Bayesian phylogenetics and phylogeography program (BPP; Yang & Rannala, 2014) which analyzes sequence alignments for closely related species with multiple loci under the multispecies coalescent (MSC) model. Using a custom python script, 200 locus files were randomly selected from the 75% locus occupancy dataset and aligned locus files in nexus format were converted to a single file for BPP input using the bpp_from_nex.py script (<https://github.com/mgharvey/misc_python>). We used the species delimitation algorithm (A10) with thetaprior = gamma(3,1500) and tauprior = gamma(3,150) and the guide tree (South, (North, Central)). Two independent runs with a burnin of 8000, sampling frequency of 10, and chain length of 5x10^5 were set. Both runs delimited the three major clades, North, Central, and South, as separate species.

**References**

Harvey, M.G. (2015). Misc_python. GitHub repository, <https://github.com/mgharvey/misc_python>

Yang, Z., & Rannala, B. (2014). Unguided Species Delimitation Using DNA Sequence Data from Multiple Loci. *Molecular Biology and Evolution, 31*(12), 3125–3135. <https://doi.org/10.1093/molbev/msu279>

**Supplemental Files**

S1_locality_details.xlsx. Excel file: Locality details for individuals included in phylogenetic and morphological analyses. Quantitative measurements of male and female characters recorded in millimeters. Highlighted rows in UCE sheet indicate specimens that also have morphological data.

S2_character_diagram_labeled.tif. Diagrammatic representation of quantitative measurements of morphological features used in morphological analyses and taxonomic description. (a) carapace length (Cl) and width (Cw), (b) labium length (LBl) and width (LBw) and sternum length (STRl) and width (STRw), (c) lengths of leg I femur (FI), patella (PI), tibia (TibI), metatarsus (MI), and tarsus (TarI), (d) lengths of leg IV femur (FIV), patella (PIV), tibia (TibIV) , metatarsus (MIV), and tarsus (TarIV), (e) male palpal tibia length (PTl) and width (PTw).

S3_all_UCE_75p_with_outgroup.pdf. Maximum likelihood phylogram based on 75% locus occupancy. Includes outgroup taxa and those removed from larger suite of analyses due to quality issues (AP_63 and AP_89). Shaded boxes correspond to major phylogenetic clades. Bootstrap support values provided at nodes.

S4_corrected_no_AP_75p_85p_95p.pdf. Maximum likelihood phylograms based on (a) 75%, (b) 85%, and (c) 95% locus occupancy. Outgroup and Baja individuals excluded. Bootstrap support values provided at nodes.

S5_ASTRAL_trees.pdf. (a) 50% (b) 70% and (c) 100% consensus species trees generated using gene trees from the 75% locus occupancy datasets. Gene trees were generated with ASTRAL-III with bootstrap resampling run with 100 pseudoreplicates in ASTRAL v5.7.8.

S6_cross_entropy_values.pdf. Results of cross-entropy validation with K values of 1 to 10 for (a) RANDSNP1 (0.59), (b) RANDSNP2 (0.55), and (c) RANDSNP3 (0.54) datasets.

S7_ancestry_proportions.pdf. Visualization of ancestry proportions estimated using sparse Non-Negative Matrix Factorization (sNMF) for RANDSNP2 (left) and RANDSNP3 (right) datasets.

S8. Excel file: Results of principal component analysis for male morphological characters. Importance of components and PC scores.

S9. Excel file: Results of principal component analysis for female morphological characters. Importance of components and PC scores.
